# Anesthesia for the dog with heartworm disease: a brief, practical review

**DOI:** 10.1186/s13071-023-05722-3

**Published:** 2023-04-28

**Authors:** Jane Quandt

**Affiliations:** College of Veterinary Medicine, University of Georgia, Athens, GA 30606 USA

**Keywords:** Heartworm disease, Anesthesia, Caval syndrome

## Abstract

Dogs with heartworm disease may present for procedures that require anesthesia. This article is a brief, practical review of anesthetic techniques for dogs with heartworm. Dogs with heartworm, such as in a shelter that undergo spay and neuter procedures, may be safely anesthetized prior to heartworm treatment. The dog presenting with caval syndrome may require emergent anesthesia for extraction of the heartworms; anesthetic drugs and potential adverse effects are discussed. The anesthetic agents that have been used are discussed.

## Background

Dogs that have tested positive for heartworm may present for procedures that require anesthesia. Prior to anesthesia, a physical examination, basic blood work and if possible thoracic radiographs and echocardiography are recommended. The concern is whether these dogs can be anesthetized safely. The severity of the disease and need for anesthesia will help determine the potential risks. A dog with minimal to mild symptoms may be able to safely undergo anesthesia. The behavior of adult heartworms during an anesthetic event is unknown. The objective of this article is to review anesthetic techniques for dogs with heartworm.

## Anesthesia in heartworm-positive dogs

Dogs may present with clinical signs including cough, dyspnea, syncope, weight loss and lethargy. Auscultation may reveal harsh lung sounds and a right apical systolic murmur over the tricuspid valve due to tricuspid regurgitation, a gallop rhythm and splitting of the second heart sound due to pulmonary hypertension [1–4].

Thoracic radiographs and echocardiography findings may include right-sided enlargement with advanced disease, enlargement, truncation and tortuosity of caudal lobar arteries, enlargement of the pulmonary artery segment and pulmonary artery infiltrates or granulomas [3, 5].

The recommended blood tests include a complete blood count, blood chemistry, urine analysis and blood coagulation profile. Abnormalities commonly seen are eosinophilia, basophilia, elevated liver enzymes, azotemia and thrombocytopenia with subclinical disseminated intravascular coagulation (DIC). These blood tests will help to understand the extent of organ system involvement [3, 4].

A recent article looked at anesthesia in heartworm-positive dogs undergoing spay or neuter procedures in a shelter [2]. All dogs had an American Society of Anesthesiologists (ASA) status of I or II; I is defined as being normal heathy patients and II are patients with mild systemic disease [6]. The dogs were heartworm (HW) positive and had additional staging with thoracic radiographs. The dogs were defined by the American Heartworm Society up to stage 2, mild disease. The protocol was cardiovascular-sparing to reduce the amount of anesthetic agents with known cardiovascular depressant effects and to select agents to maintain cardiac output and reduce pain. The protocol was acepromazine 0.01–0.05 mg/kg SC, given 1 to 2 h before surgery, and butorphanol 0.1 mg/kg IM and either meloxicam 0.2 mg/kg IM or carprofen 2 mg/kg SC, given for analgesia. Induction was with telazol 3–6 mg/kg or ketamine/diazepam 3–6 mg/kg/0.25–5 mg/kg IV with maintenance on isoflurane or sevoflurane. A lidocaine testicular block was done in male dogs. All dogs received 10 ml/kg/h IV lactated Ringer's solution (LRS). All dogs recovered from anesthesia without incident (Table 1).

**Table 1:** Anesthesia for dogs with mild, up to stage 2 heartworm disease. Suggested agents that may be used

Premedication	Induction (titrate to effect)	Maintenance
Methadone (0.1–0.2 mg/kg IM, IV)	Telazol (2–6 mg/kg IV)	Sevoflurane
Butorphanol (0.1–0.2 mg/kg IM, IV)	Ketamine (5 mg/kg with midazolam 0.25 mg/kg IV)	Isoflurane
Hydromorphone (0.1 mg/kg IM, IV)	Propofol (2–6 mg/kg IV)	IV crystalloid fluids (3–5 ml/kg/h IV)
Acepromazine (0.01–0.05 mg/kg IM, SC, IV)	Alfaxalone (1–4 m/kg IV)	
Meloxicam (0.2 mg/kg IM, SC)	Ketamine (2 mg/kg followed by propofol 3 mg/kg IV)	
Carprofen (2.0 mg/kg SC)		

The presence of adult heartworms leads to pathological damage by direct pulmonary endothelial injury, myointimal proliferation, obliterative endarteritis, an increase in vasoconstrictive mediators, thrombosis and thromboembolism leading to pulmonary hypertension [3]. This pulmonary hypertension puts a pressure load on the right heart and can lead to right ventricular dysfunction, arrhythmias and right-sided heart failure. Heartworm death and release of antigens can damage the pulmonary parenchyma, which is manifested as pneumonitis and the development of allergic pneumonitis. The most common sign of pneumonitis is cough and increased lung sounds. Crackles are due to eosinophilic inflammation and/or fibrosis. An interstitial infiltrate can be seen on radiographs [3]. Heartworm causes inflammation within the arteries and pulmonary interstitium and endothelial disruption with platelet and leukocyte adhesion. This is the rationale for the use of anti-inflammatory glucocorticoids. Endothelial damage, death of worms and fragmentation increase the risk of thrombosis and pulmonary thromboembolism (PTE) [3]. The signs of heartworm PTE include exercise intolerance, cough, cyanosis, hemoptysis, dyspnea, collapse and ascites [3]. This results in right ventricular volume and pressure overload, aggravates the hemodynamic changes due to tricuspid regurgitation and can lead to systemic congestion. The reduced preload on the left ventricle causes a reduction in cardiac output with impairment of tissue blood flow and oxygen delivery. Supraventricular and ventricular arrhythmias can be seen [3].

Off-loading the right heart is done with the inodilator, pimobendan and pulmonary vasodilation with sildenafil. Pimobendan 0.25 mg/kg is given every 12 h PO and sildenafil 1 mg/kg every 8 h PO to reduce right ventricular afterload with increased inotropy and thus improved pulmonary venous return and cardiac output. Jugular distension is indicative of increased right atrial pressure [3]. These dogs will also benefit from oxygen therapy, especially if they have severe respiratory distress.

Heartworms increase afterload, and this leads to a decrease in stroke volume. The heartworms found in the pulmonary arteries lead to vascular occlusion, which decreases the total cross-sectional area of the pulmonary vessels and is an important cause of increased pulmonary vascular resistance [7]. There is significant obstruction to blood flow, especially with dead worms that produce emboli.

Pulmonary hypertension can also facilitate the relocation of worms from the distal pulmonary arteries to the more proximal pulmonary arteries and heart producing multi-systemic caval syndrome.

## Anesthesia for dogs with caval syndrome

Caval syndrome is a life-threatening manifestation of heartworm disease that leads to forward and backward heart failure. The clinical signs are prolonged capillary refill time (CRT), tachycardia, poor pulse quality, cardiogenic shock, poor perfusion, intravascular hemolysis, pallor and pigmentation with icterus, hemoglobinuria and pale mucous membranes. Caval syndrome is characterized by severe pulmonary hypertension, decreased cardiac output, displacement and retrograde migration of adult heartworms into the main pulmonary arteries, right atrium, right ventricle and vena cava [1, 7, 8]. Worms are relocated to the tricuspid valve orifice, resulting in large volume regurgitant flow and obstruction to the right ventricular inflow along with pulmonary hypertension [3, 7]. Retrograde displacement of heartworms from the pulmonary arteries into the right heart may mechanically disrupt the tricuspid valve by becoming entangled in the valve apparatus and potentially obstructing blood flow into the right atrium and ventricle. Decreases in cardiac output as well as pulmonary arterial embolism of dead worms may play a role in the retrograde migration of heartworms from the pulmonary artery to the caudal vena cava [5, 9]. Additional clinical signs are ascites, jugular distension, heart murmur, weak femoral pulses and profound weakness. A large number of worms in the right heart entwine around and pass through the tricuspid valve apparatus. Life-threatening severe tricuspid regurgitation with poor cardiac output, intravascular red cell lysis leading to hemoglobinemia and hemoglobinuria result. There is decreased left ventricular volume and decreased left ventricular forward flow. The number of worms required to cause caval syndrome is dependent on the size of the patient, and a large worm burden can be life-threatening. Prognosis is poor without prompt heartworm extraction and should be done as soon as possible [8]. These dogs are in shock and require stabilization prior to worm removal. Place an IV catheter to give a balanced electrolyte IV fluid, such as LRS at 2–5 ml/kg/h. Intravenous fluids are necessary to treat the volume loading deficit in the right heart [8]. Blood products may include fresh frozen plasma (FFP) for the treatment of DIC, packed red blood cells (pRBC) for the treatment of anemia or whole blood to provide both plasma and red cells at 2–5 mg/kg/h IV [1]. When giving blood products decrease the rate of crystalloid fluid therapy to avoid volume overload. Vasopressors may include dopamine at 2–5 µg/kg/min or norepinephrine at 0.1–0.5 µg/kg/min IV. Once the worms have been extracted and heart function is improved, the fluid rate may be safely increased [3]. Pre-oxygenation is beneficial to help correct hypoxia [8].

Preoperative medications are clinician dependent and commonly include diphenhydramine, prednisone, pimobendan and sildenafil. Sildenafil if a selective inhibitor of cGMP-specific phosphodiesterase type 5 enzymes to treat the pulmonary hypertension. Pimobendan is a calcium sensitizer that increases right ventricular systolic function and is also an inhibitor of phosphodiesterase-3 enzyme, which affects pulmonary vasculature [9] (Table 2).

**Table 2:** Anesthesia for caval syndrome in dogs: emergency. Suggested agents that may be used

Pre-anesthesia	Premedication	Induction (titrate to effect)	Maintenance	Vasopressors Inotropes
Diphenhydramine (2 mg/kg IM) Maropitant (1 mg/kg IV)	Fentanyl (3–5 µg/kg IV, can be followed by a CRI of 5 µg/kg/h IV)	Midazolam (0.2 mg/kg IV)	Sevoflurane at a minimal concentration	Dopamine (2–5 µg/kg/min)
Dexamethaxone (0.2 mg/kg IV once if not on NSAIDs)	Butorphanol (0.2 mg/kg IV)	Propofol (2–4 mg/kg IV**)**	Isoflurane at a minimal concentration	Norepinephrine (0.1–0.5 µg/kg/min IV)
Place IV catheter and give crystalloid fluids (3–5 ml/kg/h IV) Pre-oxygenate	Hydromorphone (0.1 mg/kg IV)	Etomidate (1–2 mg/kg IV, must be preceded by midazolam)		A positive inotrope Dobutamine (5–10 µg/kg/min IV to improve cardiac contractility)
May need FFP for clotting factors or pRBC for anemia, give via dedicated IV catheter Decrease crystalloids to avoid volume overload	Methadone (0.1 mg/kg IV**)**	Alfaxalone (1–4 mg/kg IV) Lidocaine (2 mg/kg IV can be followed by a CRI of 3 mg/kg/h IV**)**		

Treatment of choice for extraction is via a right jugular venotomy [1, 3]. A local block is done using lidocaine 1–2 mg/kg over the right jugular vein, which can be ligated permanently or temporarily occluded with umbilical tape above the venotomy site. The venotomy is performed, and the retrieval device is passed down the jugular vein to the vena cava and right atrium. Guidance during the extraction procedure can be done with echocardiography, transthoracic or transesophageal to identify worms in the right heart chambers and/or pulmonary artery, and fluoroscopy [3]. Echocardiography reveals many short parallel hyperechoic lines mimicking “equal signs” that move back and forth with each heartbeat as a large bundle between the right atrium and the inflow of the right ventricle [5, 9]. Transesophageal echocardiography (TEE) has been used for visualization during worm retrieval. The TEE allowed for clear visualization of the heartworms in the right heart chamber and in the pulmonary artery and also the tip of the forceps when opening and closing the jaws [5]. Fluoroscopy is essential to ensure the forceps are in an appropriate and safe position as TEE does not allow visualization of the forceps in distal sites. TEE does offer the advantage of allowing direct information related to location and number of worms, facilitation of forceps deployment and confirmation the worms have been removed from the vessels and right heart. TEE provides intraoperative real time functional information [5]. Once the retrieval device is properly positioned, worms are grasped and removed with careful pulling as the worms can be wrapped around the chordae tendinae. Do not use excessive force or pull too hard as this can result in worm laceration, release of antigen and subsequent anaphylaxis. Macerating worms results in a massive antigen release, pulmonary vasoconstriction and DIC [3]. Successful outcomes are between 50 and 67% [9]. Potential adverse effects during heartworm extraction include cardiac arrest, hypotension, DIC and inability to remove all the worms [1]. Once worms are removed the right heart failure abates rapidly. When the worms are removed, the tricuspid regurgitation disappears, cardiac output increases, and right atrial pressure decreases.

The extraction is done under general anesthesia or deep sedation. One study used midazolam, diazepam, fentanyl, etomidate and lidocaine IV with maintenance on sevoflurane. In this 2010 retrospective study, 6 of 21 dogs died. Two dogs died during the procedure of cardiac arrest secondary to unresponsive bradycardia; normal sinus rhythm was restored but the dogs did not regain consciousness and were subsequently euthanized. Three dogs died 24 h after surgery, and one dog died 72 h later. Two of these dogs had DIC before surgery, and all four were hypotensive during the surgery. One dog developed cardiac arrest that required resuscitation during the surgery and was later euthanized, one developed seizures following the surgery and was euthanized, one died of aspiration pneumonia, and one dog developed progressive DIC. Fourteen of 21 dogs survived to discharge; they had uneventful anesthesia and recovery. Dogs with high serum ALT had a lower survival rate. Based on the model, high ALT and heartworms in the pulmonary artery predicted a survival rate of 0% in this small sample size of 18 dogs. Mortality rates of 30 to 40% with a poor to guarded prognosis are found even with extraction [1] (Table 3).

**Table 3:** Deep sedation protocol [10]

Premedication	Induction	Maintenance	Analgesia
Dexmedetomidine (5 µg/kg IV) Butorphanol (0.4 mg/kg IV) Lidocaine (2 mg/kg IV followed by a lidocaine CRI of 0.04 mg/kg/min IV)	Etomidate (0.1 mg/kg IV)	Sevoflurane with a vaporizer setting at 0.5%. A local block of lidocaine/bupivacaine done over the surgical site. Dexmedetomidine at 1 µg/kg IV given if there is any movement. End of procedure-dexmedetomidine reversed with atipamezole 0.05 mg/kg IM	Buprenorphine (0.01 mg/kg IV)

Anesthetic agents that have been used include midazolam, diazepam, fentanyl, etomidate, midazolam and fentanyl CRIs and sevoflurane [1]. Other anesthetics have included atropine, benzodiazepines, fentanyl, lidocaine, propofol and inhalants. The use of alpha two agonists is controversial. Potential advantages of dexmedetomidine are prevention of catecholamine-induced arrhythmias and inhalant anesthetic sparing effect. The use is controversial in the cardiac compromised patient as alpha two agonists cause vasoconstriction of vascular smooth muscle; this increases systemic and pulmonary pressure and causes a reflex bradycardia. There is increased left ventricular afterload, increased cardiac work and reduced cardiac output and oxygen delivery.

The use of opioids and benzodiazepines is preferred as they have minimal cardiovascular effects, and the opioid-mediated bradycardia can be treated with anticholinergics if necessary. Alpha two agonists can lead to a severe reduction in cardiac output at doses ≥ 5 µg/kg. Acepromazine is controversial as the alpha-1 adrenergic-receptor-antagonist-mediated decrease in systemic vascular resistance can lead to decreases in blood pressure that is long lasting and irreversible. Etomidate and fentanyl, combined with a benzodiazepine, have minimal effects on cardiac contractility and systemic vascular resistance and are useful for induction to maintain cardiac output. Invasive blood pressure monitoring is recommended [4].

A case report discussed minimally invasive surgical extraction done successfully in two dogs [10]. Extraction was done under deep sedation with dexmedetomidine 5 µg/kg, butorphanol 0.4 mg/kg and lidocaine 2 mg/kg IV and lidocaine CRI 0.04 mg/kg/min IV. Etomidate 0.1 mg/kg IV was given followed by intubation. Anesthesia was maintained on sevoflurane with a vaporizer setting at 0.5%. A local block of lidocaine/bupivacaine was done over the surgical site. Dexmedetomidine at 1 µg/kg IV was given if there was any movement. Occasional multifocal PVCs were observed during heartworm retrieval. At the end of the procedure, dexmedetomidine was reversed with 0.05 mg/kg IM atipamezole. Analgesia was provided with buprenorphine at 0.01 mg/kg IV. This sedation technique provided a satisfactory outcome in these two dogs [10].

In some instances, following general anesthesia the heartworms may migrate into the pulmonary arteries, making extraction impossible [1]. These cases are then treated with adulticide. A recent paper looked at the resolution of caval syndrome that occurred during initial hemodynamic stabilization of the dogs [9]. This paper discussed five dogs that underwent stabilization to improve their hemodynamics prior to heartworm extraction. During the stabilization treatment these dogs had a rare and unanticipated spontaneous resolution of their caval syndrome. All dogs had a 3 to 5/6 auscultated right apical systolic murmur. Dogs received IV crystalloid fluid therapy, 60–100 ml/kg/day, or 10 ml/kg boluses to treat hypotension, pimobendan 0.25–0.3 mg/kg PO every 12 h, sildenafil 1.1–2.8 mg/kg PO every 8 h, prednisone 0.5–0.7 mg/kg PO every 12 to 24 h or dexamethasone-SP 0.15 mg/kg IV every 24 h, clopidogrel 2.9 mg/kg PO every 24 h in one dog and oxygen therapy. The next morning echocardiography showed no heartworm signs, indicating that blood flow had carried the heartworms back into the pulmonary circulation. The extraction procedure was cancelled, and dogs were treated with adulticide as per the American Heartworm Society guidelines and continued on pimobendan and sildenafil [9]. Excessive fluid therapy should be avoided as that can impair cardiac output by worsening tricuspid regurgitation. Heartworm extraction is necessary for most dogs with caval syndrome, but medical stabilization may result in spontaneous resolution of caval syndrome.

Following heartworm extraction medical treatment should include an adulticide with follow-up as the extraction cannot reach into the pulmonary arteries to retrieve worms located there [8]. The American Heartworm Society recommends a 4-week course of doxycycline followed by a month of rest and then the administration of the three-injection adulticide treatment [11]. These dogs can have a good long-term prognosis [1, 3]. Postoperative mortality is still possible following successful worm retrieval and recovery from anesthesia due to pulmonary embolism [5]. To reduce the risk of embolic events, consider exercise restriction and the administration of glucocorticoids, anticoagulants and vasodilators [5].

## Four caval syndrome case examples

Four dogs presenting with caval syndrome underwent anesthesia for worm extraction. All the dogs were rated ASA status IV E, which are patients with severe systemic disease that is a constant threat to life and E constitutes an emergency [6]. All four dogs had successful worm retrieval and lived.

### Case 1

A bulldog weighing 23.8 kg with a PCV 35% and TS 6.0 g/dl underwent emergency heartworm extraction. Preoperative medication included maripitant 1 mg/kg and dexamethasone SP 0.15 mg/kg, both given IV. Premedication consisted of fentanyl 3 µg/kg IV, midazolam 0.2 mg/kg IV and lidocaine 2 mg/kg IV. Propofol 0.8 mg/kg was given IV to allow for intubation. A fentanyl 5 µg/kg/h IV and a lidocaine 3 mg/kg/h IV constant rate infusion (CRI) were maintained for the duration of the worm extraction; additional anesthesia was provided by isoflurane. Fluid therapy was LRS at 3 ml/kg/h IV. Recovery was uneventful.

### Case 2

An 8-year-old Maltese mix weighing 4 kg, with a PCV 32% and TS 5.4 g/dl, had heartworm extraction. Diphenhydramine 2 mg/kg was given IM prior to anesthesia. Anesthesia consisted of butorphanol 0.2 mg/kg IV, midazolam 0.2 mg/kg IV and etomidate 1 mg/kg IV to allow for intubation with maintenance of sevoflurane. Fluid therapy was LRS 2 ml/kg/h IV, and due to anemia 45 ml of pRBCs was given IV. Lidocaine at a 2 mg/kg loading dose and then 3 mg/kg/h IV CRI was added. The dog had severe hypotension; a fluid bolus at 10 ml/kg IV was given followed by a second bolus of 5 ml/kg IV. Dobutamine 5 µg/kg/min up to 10 µg/kg/min IV was given to try improve blood pressure. At one time during the procedure no peripheral pulses were palpated and there were no blood pressure readings. Blood pressure readings returned once the worms were removed and the second fluid bolus given. In recovery, flumazenil 0.1 mg IV, naloxone 0.12 mg IV, a second dose of flumazenil IV and a second dose of 0.08 mg naloxone IV were given to reverse the anesthesia and allow extubation.

### Case 3

A 6-year-old Chihuahua weighing 3.5 kg with a PCV 21% and TS 5.2 g/dl underwent heartworm extraction. Anesthesia consisted of hydromorphone 0.1 mg/kg IV, midazolam 0.25 mg/kg IV, maripotant 1 mg/kg IV, propofol 2 mg/kg IV and isoflurane. LRS was given as a 15 ml bolus before induction and then 5 mg/kg/h IV during the procedure. The anesthetic event and recovery were smooth. A total of 13 worms were extracted.

### Case 4

Bruce was a 7-year-old MI, 6.8 kg dog, not vaccinated, not on HW preventive, full of fleas with a PCV 21%. He presented in right heart failure due to caval syndrome. The dog had been given steroids, diphenhydramine, cerenia, sildenafil, pimobendan and pRBC transfusion as the initial PCV was 15%. He was also given (nitenpyram) Capstar™ rectally to treat the flea infestation. The dog was induced in the interventional radiology suite. The anesthetic drugs were given IV and included fentanyl, midazolam, lidocaine, etomidate and maintenance on sevoflurane. Fluid therapy was LRS at 2 ml/kg/h IV along with a lidocaine CRI of 2 mg/kg loading dose and 3 mg/kg/h IV. Twenty-three worms were extracted, and the dog did well.

## Conclusion

It is recommended to do a pre-anesthetic CBC, chemistry, UA and potentially coagulation panel in dogs that are heartworm-positive prior to anesthesia to assess degree of organ involvement. Thoracic radiographs and echocardiographic examination will provide information on the degree of heart compromise. Anesthesia should be done with a cardiovascular-sparing protocol. Caval syndrome is a life-threatening condition and requires heartworm extraction. The administration of blood products and vasopressors may be required with caval syndrome. Consider giving diphenhydramine and dexamethasone prior to heartworm extraction to help prevent an anaphylactic response to macerated or torn worms. Pimobendan and sildenifil are given to improve right ventricular function and treat pulmonary hypertension. Dogs with heartworm disease can be safely anesthetized but require diligent monitoring during and after the anesthetic episode.

## Data Availability

Not applicable.

## Abbreviations

DIC: Disseminated intravascular coagulation
IV: Intravenous
IM: Intramuscular
ASA: American Society of Anesthesiologists
SC: Subcutaneous
HW: Heartworm
LRS: Lactated Ringer's solution
FFP: Fresh frozen plasma
pRBC: Packed red cells
NSAIDs: Nonsteroidal anti-inflammatory drugs
TEE: Transesophageal echocardiography
CRI: Constant rate infusion
PVC: Premature ventricular contraction
PO: Per os
PCV: Packed cell volume
TS: Total solids
PTE: Pulmonary thromboembolism
CRT: Capillary refill time
